# Next-Generation Sequencing of Disseminated Tumor Cells

**DOI:** 10.3389/fonc.2013.00320

**Published:** 2013-12-31

**Authors:** Elen K. Møller, Parveen Kumar, Thierry Voet, April Peterson, Peter Van Loo, Randi R. Mathiesen, Renathe Fjelldal, Jason Grundstad, Elin Borgen, Lars O. Baumbusch, Bjørn Naume, Anne-Lise Børresen-Dale, Kevin P. White, Silje Nord, Vessela N. Kristensen

**Affiliations:** ^1^Department of Genetics, Institute for Cancer Research, Oslo University Hospital Radiumhospitalet, Oslo, Norway; ^2^K.G. Jebsen Center for Breast Cancer Research, Institute for Clinical Medicine, Faculty of Medicine, University of Oslo, Oslo, Norway; ^3^Centre for Human Genetics, Department of Human Genetics, University Hospital Leuven, KU Leuven, Leuven, Belgium; ^4^Single-Cell Genomics Centre, Wellcome Trust Sanger Institute, Hinxton, UK; ^5^Institute for Genomics and Systems Biology, Department of Human Genetics, The University of Chicago, Chicago, IL, USA; ^6^Cancer Genome Project, Wellcome Trust Sanger Institute, Hinxton, UK; ^7^Department of Human Genetics, VIB and KU Leuven, Leuven, Belgium; ^8^Department of Oncology, Division of Surgery and Cancer Medicine, Oslo University Hospital Radiumhospitalet, Oslo, Norway; ^9^Department of Pathology, Oslo University Hospital Radiumhospitalet, Oslo, Norway; ^10^Institute for Clinical Medicine, Faculty of Medicine, University of Oslo, Oslo, Norway; ^11^Department of Clinical Molecular Biology (EpiGen), Medical Division, Akershus University Hospital, Lørenskog, Norway

**Keywords:** single tumor cell sequencing, disseminating tumor cells, circulating tumor cells, tumor heterogeneity, clonal evolution

## Abstract

Disseminated tumor cells (DTCs) detected in the bone marrow have been shown as an independent prognostic factor for women with breast cancer. However, the mechanisms behind the tumor cell dissemination are still unclear and more detailed knowledge is needed to fully understand why some cells remain dormant and others metastasize. Sequencing of single cells has opened for the possibility to dissect the genetic content of subclones of a primary tumor, as well as DTCs. Previous studies of genetic changes in DTCs have employed single-cell array comparative genomic hybridization which provides information about larger aberrations. To date, next-generation sequencing provides the possibility to discover new, smaller, and copy neutral genetic changes. In this study, we performed whole-genome amplification and subsequently next-generation sequencing to analyze DTCs from two breast cancer patients. We compared copy-number profiles of the DTCs and the corresponding primary tumor generated from sequencing and SNP-comparative genomic hybridization (CGH) data, respectively. While one tumor revealed mostly whole-arm gains and losses, the other had more complex alterations, as well as subclonal amplification and deletions. Whole-arm gains or losses in the primary tumor were in general also observed in the corresponding DTC. Both primary tumors showed amplification of chromosome 1q and deletion of parts of chromosome 16q, which was recaptured in the corresponding DTCs. Interestingly, clear differences were also observed, indicating that the DTC underwent further evolution at the copy-number level. This study provides a proof-of-principle for sequencing of DTCs and correlation with primary copy-number profiles. The analyses allow insight into tumor cell dissemination and show ongoing copy-number evolution in DTCs compared to the primary tumors.

## Introduction

Tumor cell dissemination to distant sites is one prerequisite for metastasis, and the presence of single tumor cells in bone marrow (BM) (disseminated tumor cells, DTCs) and/or circulating tumor cells (CTCs) in blood, has been shown to be a strong prognostic factor in early breast cancer (1–4). The entire process behind tumor progression and development of metastasis is not fully understood. The release of tumor cells from the primary site to the circulation (5) and gain of additional genetic alterations has by some investigators been postulated to occur in parallel to the formation of the primary tumor. Others believe they descend from certain subclones present in a heterogeneous primary tumor. These two models are referred to as parallel and step-wise progression, respectively (6). There is growing evidence for primary tumor heterogeneity, based both on phenotypical and genotypical differences within the tumor (7–9). When comparing the expression of single genes and proteins between primary tumor and metastatic tissue, disparities have been observed in a subset of the samples (10), becoming even more evident when performing genome-wide analyses (11, 12). Differences between the primary tumor and overt metastases may be caused by further tumor evolution at distant sites and/or different disseminating and metastatic potential between subclones in a heterogeneous primary tumor.

Adjuvant systemic treatment after primary surgery of breast cancer has improved survival markedly over the last decade (13). However, depending on the cancer subtype, the systemic treatment does not cure more than 30–60% of the patients with minimal residual disease (14). This may be caused by general resistance to the treatment administered or treatment insufficiency because of treatment-related differences between the crude primary tumor and the DTCs. Analysis of genomic changes at the single-cell level may reveal the underlying composition of the cells that have disseminated, and from which subclone they originate (7). In this respect, the acquisition of methods for in-depth single-cell analysis is important to obtain the necessary knowledge of the tumor cell dissemination and metastatic process and in turn, it is crucial for targeted treatment of DTCs.

Today’s sequencing techniques can discover all classes of DNA mutation, but do not allow us to directly study the small amounts of DNA obtained from a single cell. Therefore whole-genome amplification (WGA) is required to enable the analysis of a single cell’s genome. Importantly various artifacts created during WGA may affect downstream detection of reliable genuine genetic variants. WGA of single-cell DNA can be based either on multiple displacement amplification (MDA), polymerase chain reaction (PCR), or methods combining principles of both. Furthermore, the amount and nature of artifacts can vary significantly between different WGA methods. Although pure MDA-based WGA methods appear to be the method of choice for typing single-nucleotide variants (15–19), WGA-methods involving PCR seem to preserve single-cell DNA-copy number changes more accurately during the amplification process and may be used for single-nucleotide variant detection as well (20–22).

Previously genomic rearrangements in DTCs have been studied using low resolution technologies, e.g., comparative genomic hybridization (CGH), array comparative genomic hybridization (aCGH), and fluorescence *in situ* hybridization (FISH) (23–25). Analysis of copy number changes in DTCs by aCGH only allows us to identify genomic modifications larger than two megabases due to the difficulty in interpreting the inherently noisy single-cell WGA data and the limited array-resolution genome wide (25). In contrast, sequencing technology enables us to study copy-number alterations in more detail by focal read depth analyses (7, 26) and to compute a digital B-allele frequency (BAF) (22). The latter can not only corroborate DNA-copy number changes, but also reveal copy neutral loss of heterozygosity (cnLOH).

The aim of this study was to investigate whether whole-genome sequencing of WGA DNA obtained from single (micromanipulated) DTCs could provide reliable data of genetic changes, as well as to provide a proof of principle that it is possible to compare the copy-number landscape of a DTC to that of its primary tumor. In addition, we wanted to see if we could recapitulate the copy number changes from previous aCGH results, and in even greater detail gain information of the genetic alterations in a single cell.

## Materials and Methods

### Patient material

Bone marrow and primary tumor were analyzed from two localized breast cancer patients included in the Oslo Micrometastasis (MicMa) project, classified as pT2pN0M0 and pT2pN3M0 (3). Both patients had estrogen receptor (ER) and progesterone receptor (PR) positive tumors, both were human epidermal growth factor receptor 2 (Her2) negative. Tumors were fresh frozen at −80°C and DNA was extracted using an ABI 341 Nucleic Acid Purification System (Applied Biosystems, Carlsbad, CA, USA) according to the manufacturer’s protocol. BM was aspirated from posterior iliac crests (bilaterally) at the time of surgery, followed by isolation of mononuclear cells and by preparation of cytospins for detection of DTCs by immunocytochemistry (ICC) (25).

### Detection, micromanipulation, and DNA amplification of DTCs

**Figure 1:**
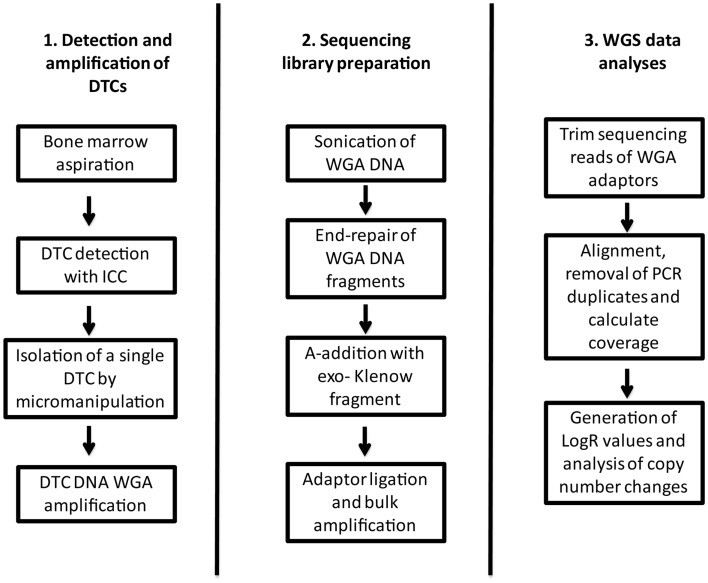
**Flow chart describing the workflow from collection and detection of the DTC, to library preparation and data analyses**.

All methods are described in detail in Mathiesen et al. (25). In brief, detection of DTCs was performed by ICC staining of the cytospins, followed by isolation of single tumor cells by micromanipulation (see flow chart in Figure 1). The cell was deposited in an optical lid closed with an Eppendorf PCR tube, centrifuged for 10 s, and stored at −20°C. Amplification of DNA for the DTC was performed using GenomePlex^®^ Single-Cell Whole-Genome Amplification Kit (Sigma-Aldrich, St. Louis, MO, USA). Cell lysis, fragmentation, and library preparation were all done according to the manufacturer’s instructions. DNA amplification was performed using Sigma-Aldrich amplification master mix and Titanium Taq DNA polymerase (BD Biosciences Clontech, Heidelberg, Germany).

### Array comparative genomic hybridization of DTCs

Agilent’s Human Genome 244k CGH microarray (Agilent Technologies, Santa Clara, CA, USA) was used to detect copy-number alterations in WGA DNA from the DTCs. Scanned microarrays were preprocessed and linear normalized using Feature Extraction (Agilent Technologies, Santa Clara, CA, USA) (GEO accession number GSE27574). The piecewise constant fitting (PCF) algorithm was used to detect chromosomal gains and losses (minimum amount of probes for segments, *k*_min_, was set to 25 and the penalty parameter, γ, was set to 60) (27). To avoid outliers, a lowest detectable value was set and lowest limit on a log2 scale was set to −2. The zero line was subsequently redefined to give an average value of 0 across all chromosomes. The procedure is thoroughly described in Mathiesen et al. (25). Additional GC-correction of the data was executed here.

### Whole-genome sequencing library preparation of DTCs

Whole-genome amplification DNA (1 μg) was sheared using the S220 Focused-ultrasonicator (Covaris, Woburn, MA, USA) followed by end repair using the End-It™ DNA End-Repair Kit (Epicenter, Madison, WI, USA) (Figure 1). The end-repair master mix was made as specified by the manufacturer, mixed with sheared DNA, and incubated at room temperature for 45 min. Subsequently each sample underwent A-base addition using an A-addition master mix and incubated at 37°C for 30 min. The A-addition master mix consisted of 5 μl 10× NEBuffer 2 (New England Biolabs, Ipswich, MA, USA), 10 μl dATP (1 mM) (Sigma-Aldrich, MO, USA), and 3 μl Klenow [3′ → 5′exo-] (Thermo Scientific). Following A-base addition, adaptors were ligated onto the DNA fragments by an adaptor ligation master mix and each sample was incubated at 16°C for 20 min. Adaptor ligation master mix was made out of 5 μl 10× T4 Ligase Buffer (New England Biolabs, Ipswich, MA, USA), 5 μl adaptor (50 μM), and 2 μl T4 DNA Ligase (2000 U/μl) (New England Biolabs, Ipswich, MA, USA) to each sample. After adaptor ligation the sample libraries were PCR-amplified for 12 cycles. Amplification master mix consisted of 2.5 μl universal primer, 2.5 μl index primer, and 250 μl 2× iProof High-Fidelity Master Mix (Bio-Rad Laboratories, Hercules, CA, USA). The amplification master mix (255 μl) and MQ water was added to 125 ng of the template to a total volume of 500 μl, spread across five wells at a reaction volume of 100 μl. After each step described above, samples were column cleaned using the QIAquick PCR Purification Kit (Qiagen, Valencia, CA, USA). Following amplification, library quality assessment was done using Bioanalyzer (Agilent Technologies, Santa Clara, CA, USA). Further cleaning was performed using Agencourt AMPure XP (Beckman Coulter, Brea, CA, USA). Samples were sequenced on HiSeq 2000 (Illumina, San Diego, CA, USA) with half a sequencing lane, over two lanes, to a total of one lane per sample. Sequencing data can be obtained by contacting the authors.

### Mapping of DTC-sequences

Whole-genome amplification-adapter sequence may contaminate single-cell sequencing reads. Hence, the sequencing reads were first investigated for recurrent bases at each position across the reads. All reads were trimmed by 32 bases to remove WGA-adapter sequence contamination, and were subsequently aligned to GRCh37 human reference genome using BWA (28). The resulting BAM files were further processed by removal of PCR duplicates and genomic coverage was calculated using Picard (http://picard.sourceforge.net/) and Bedtools (29) respectively (Figure 1).

### Copy-number analysis and SNP BAF-typing of single DTCs

Copy-number profiles and SNP B-allele fraction profiles were determined using a novel pipeline based on principles from Voet et al. (22) and Baslan et al. (26).

In order to conduct focal read depth analysis, genomic bins were first defined by generating artificial reads of a length equal to the single-cell reads (69 bases after trimming) from every base in the human genome and mapping them back to the reference genome using BWA. Reads mapping at multiple loci were discarded resulting in “uniquely” mappable positions. Subsequently, the human genome was divided into non-overlapping bins of 50 kb unique positions (26) resulting in physical bin sizes of 51.5 kb on average (1.8 kb SD, when 1% of the top bins were removed). To each bin’s single-cell read-count a value of one was added, and bins with a %GC-content of <28% were discarded. The log*R*-values for these non-overlapping variable bins were subsequently computed by dividing the read-count of a given bin by the average read-count of the bins genome wide. The log*R*-values were corrected for %GC-bias using a Loess fit in *R*, and were normalized according to the median of the genome-wide log*R*-values (Figure 1). Corrected log*R*-values were segmented using PCF (the penalty parameter, γ, was set to 25) (27). Integer DNA-copy number was estimated as 2^log^*^R^* × Ψ, where the average ploidy, Ψ, of the tumor cell was estimated based on the log*R* value of a large reference region with known DNA-copy number without large copy-number aberrations (22).

To perform SNP genotyping from the sequencing data, we used known SNP positions from dbSNP (version 135) and calculated a SNP BAF for each SNP covered by at least eight reads (22).

### Copy-number analysis of primary tumors

Primary tumor DNA was analyzed for copy-number aberrations using Genome-Wide Human SNP array 6.0 (Affymetrix, Santa Clara, CA, USA). Raw data was normalized by HapMap using Affymetrix Power tools and copy-number aberrations were analyzed using the allele-specific copy-number analysis of tumors (ASCAT) (30). ASCAT germline prediction was performed for MicMa003, due to noisy blood SNP-CGH data, while MicMa083 was analyzed with matched germline data. Segmentation of the data was performed by the allele-specific piecewise constant fitting (asPCF) algorithm (27). SNP-CGH data can be obtained by contacting the authors.

## Results

In this study we have sequenced WGA DNA from two DTCs (DTC003 and DTC083) derived from the BM of two non-metastatic breast cancer patients at the time of diagnosis. We have optimized the sequencing protocol and data analysis for DTCs, as well as compared copy-number alterations in the single cells and the corresponding primary tumor.

### Whole-genome sequencing of DTCs

DTC003 and DTC083 were whole-genome sequenced to 14.5 and 10.45 Gb, respectively. Following mapping and PCR duplicate removal, the attained breadth of coverage was 33.9 and 38.7% of the human reference genome, while the depth of coverage was 2.92× and 2.03× (when compared to the size of the human reference genome) and 8.61× and 5.25× (when compared to the breadth of covered bases), respectively. This allowed us to study the copy-number landscape in combination with the SNP B-allele fractions.

### Copy-number analysis comparing whole-genome sequencing to aCGH

**Figure 2:**
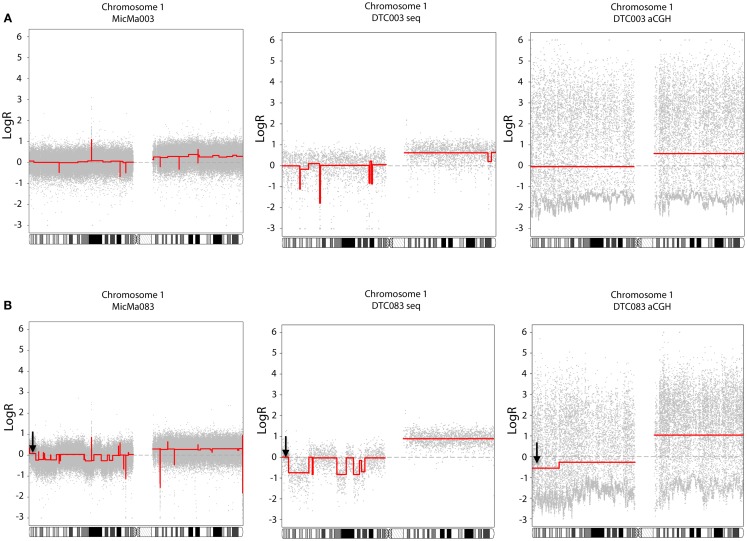
**Comparison of copy-number data of DTCs generated from whole-genome sequencing (middle side) and aCGH (right side), with the primary tumor as a control (left side)**. The piecewise constant fitting algorithm was used to generate segments with gamma = 25 and *k*_min_ = 5 for the sequencing and primary tumor data, and gamma = 60 and *k*_min_ = 25 for the aCGH data. **(A)** DTC003: gain of chromosome 1q was observed with both technologies, where a focal deletion on 1p was only detected in the DTC sequencing data, and not recapitulated in either the DTC aCGH or primary tumor profile. **(B)** DTC083: copy-number alteration on chromosome 1 shows that aCGH data do not recapitulate the smaller deletion on the 1p loci, however both technologies capture the 1q whole-arm amplification. A false aberration is found in the aCGH data (marked with a black arrow).

To analyze the copy number changes in the DTCs, log*R* ratios were derived from local sequencing depth of the sample using non-overlapping variable windows (mean size 51.5 kb), without applying a deep-sequenced reference sample as reported previously (22), thereby also circumventing log*R* anomalies induced by CNVs present in the reference sample. The normalized log*R*-values were segmented using PCF and copy-number profiles were generated (described in detail in Materials and Methods). Results were compared to previously reported aCGH copy-number analyses of the same DTCs (25). Some differences and similarities of the two approaches are illustrated in Figure 2, where PCF parameters were set based on amount of noise in the data. A copy-number profile derived from non-WGAed DNA extracted from the primary tumor (see also below) was in addition applied as a control. DTC003 had a whole-arm amplification of 1q observed by aCGH and sequencing, which is also present in the non-WGA primary tumor profile, corroborating its authenticity (Figure 2A). Additional focal deletions were seen in the sequencing data, but were not observed in either the DTC aCGH or primary tumor profile. In contrast, DTC083 (Figure 2B) shows several small deletions on chromosome 1p, which are endorsed by the MicMa083 profile (see also Figure 4A), but which are only captured by sequence data analysis, not by aCGH analysis of the same single-cell WGA-product. The latter aCGH approach only captures a larger deletion on 1p and a whole-arm 1q gain in DTC083. Figure 2B also shows a false positive deletion (indicated with an arrow) on 1p detected in the aCGH data. Similar findings were seen on other chromosomes (Figure S1 in Supplementary Material).

### Copy-number analysis comparing whole-genome sequencing of DTC to SNP-CGH (subclone) analysis of the primary tumor

**Figure 3:**
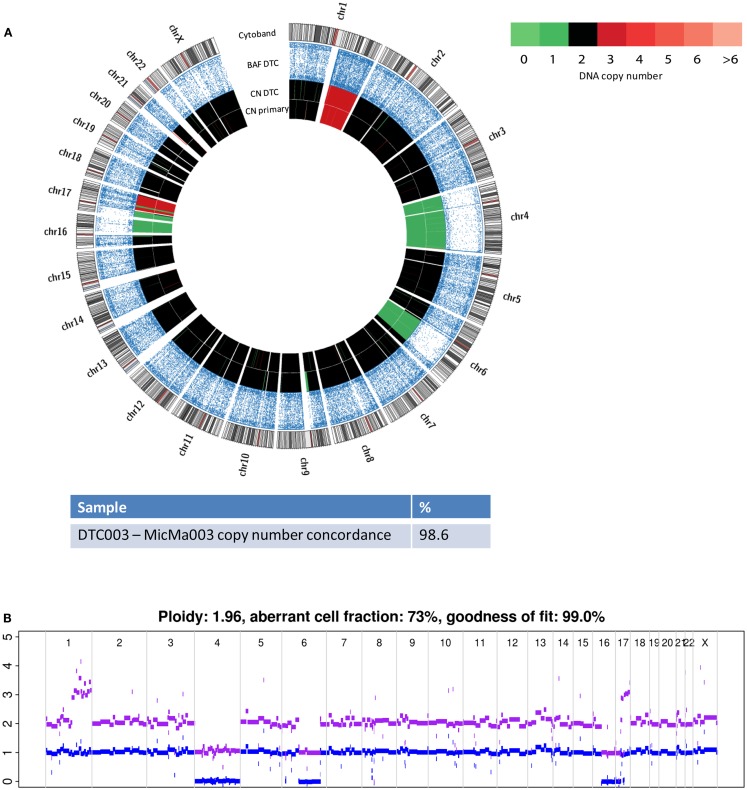
**Copy-number changes were analyzed from sequencing data from the DTC003 and SNP-CGH data from the corresponding primary tumor, MicMa003**. **(A)** Comparison of aberration pattern in the DTC and primary depicted by Circos plots. BAF reveals allelic loss and copy-number alterations of the DTC003 showed ~99% concordance with its primary tumor. **(B)** SNP-CGH profile of primary tumor shows few subclonal alterations. Clonal amplifications and deletions were observed.

The two primary tumors (MicMa003 and MicMa083) corresponding to DTC003 and DTC083 were analyzed using SNP-CGH (Materials and Methods) and their copy-number profiles were generated using the ASCAT algorithm followed by allele-specific segmentation by the PCF algorithm (27, 30). Copy-number analysis showed few aberrations in DTC003, including a monosomy 4, large-scale deletions on chromosomes 6, 16, and 17, and large-scale amplifications on chromosomes 1 and 17 (Figure 3A). The primary tumor, MicMa003, showed a SNP-CGH copy-number profile that is ~99% concordant to the corresponding DTC. No major subclonal changes at the copy-number level were detected in the primary tumor by ASCAT (Figure 3B).

**Figure 4:**
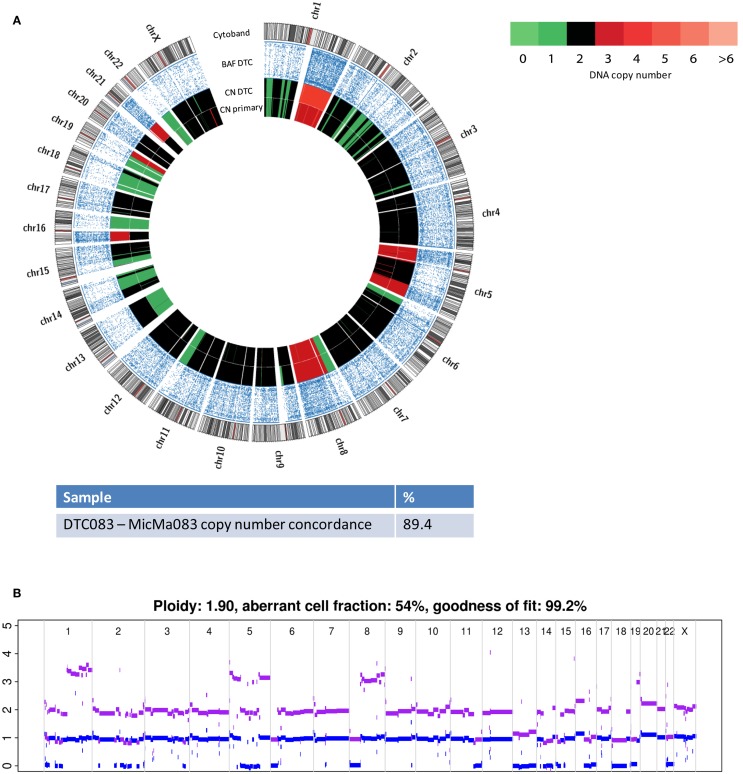
**Copy number changes were analyzed from sequencing data from the DTC083 and SNP-CGH data from the corresponding primary tumor, MicMa083**. **(A)** Circos plots reveal aberration pattern differences between the DTC and primary tumor, with ~89% concordance in the copy number changes. BAF show cnLOH on chromosome 13 in the DTC where the primary tumor had a subclonal deletion. Amplification of chromosome 21 seems to be novel in the DTC. **(B)** ASCAT profile of primary tumor describes several subclonal alterations, such as amplification on chromosome 1q and 16p, and deletion of chromosome 13.

**Figure 5:**
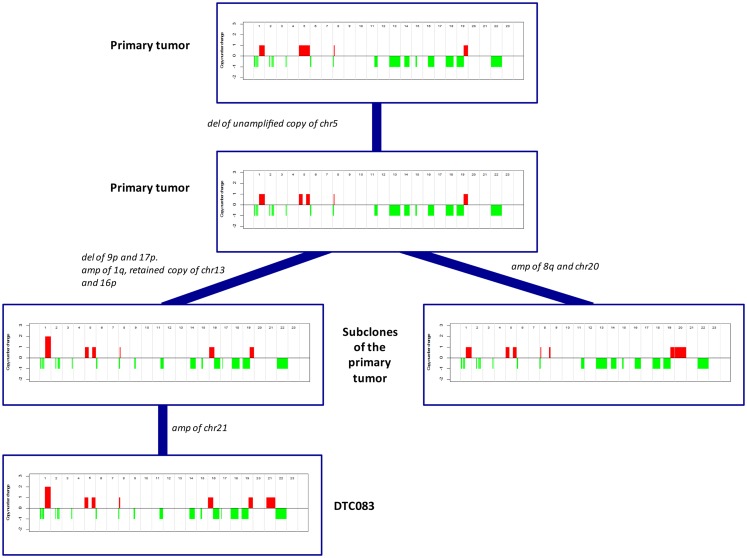
**A proposed and animated model of how the tumor evolution has occurred in MicMa083 at the copy-number level**. Aberrations change from clonal to subclonal, where the DTC could descend from one of the primary subclones.

Similarly to its corresponding DTC, the more highly aberrant primary tumor, MicMa083, showed multiple alterations over the whole genome (Figure 4A). At first glance, the primary tumor demonstrated a SNP-CGH copy-number profile that was ~89% concordant to its corresponding DTC. However, when studied in more detail, the primary tumor copy-number profiles indicated the existence of subclonal changes (Figure 4B), from which DTC083 likely descents (Figure 4A). For instance a deletion of one of the alleles of chromosome 13 was observed as subclonal in the primary tumor, while the DTC had a cnLOH (copy neutral LOH). Similarly, DTC083 showed a DNA-gain of chromosome 16p that was observed as subclonal (Figure 4B) in the primary tumor, while a trisomy 21 in the DTC was not apparent in the primary. A putative chromothripsis event on chromosome 2 was recapitulated in both the primary tumor and the DTC (Figure 4A). These clonal and subclonal events provide evidence of a tumor evolution at the copy-number level. Taken together, these results indicate that the DTC could be derived from a subclone of the primary tumor. Figure 5 illustrates animated genomic changes explaining a possible tumor progression model for MicMa083.

## Discussion

Single-cell analyses have become an important tool in analysis of tumor heterogeneity (7), as well as in detection and characterization of the genomic complexity of CTCs (21) and DTCs. So far, genomic alterations of DTCs have only been detected by aCGH and/or FISH methods (24, 25, 31). In this paper, we demonstrate that whole-genome sequencing of single DTCs is feasible and may provide information about high-resolution genetic changes, as well as copy-number neutral events.

In contrast to multi-cell analysis, investigation of genomic changes in a single cell requires WGA due to the small amount of DNA acquired from a single tumor cell. Various sequence biases introduced by WGA have been reported and it may be difficult to distinguish between the true genomic variation of the cell and allelic amplification introduced by the amplification process (7, 22, 32). Consequently, this can lead to difficulties in detection and interpretation of true copy-number changes. Creating non-overlapping, uniquely mapping genomic bins, and calculating the read count for each bin provided high-resolution copy-number data following WGA-specific GC-bias adjustment. In the aCGH analysis by Mathiesen et al. a moving Log*R* average was calculated to avoid false aberrations caused by the amplification (25). The study demonstrated how single-cell aCGH from WGA DNA could be used to detect genomic aberrations in DTCs from BM, however the sparse coverage of the genome, due to the WGA, resulted in noisy data with a systematic fluctuation in probe values. Segmentation of the aCGH was also affected by the noise, making it problematic to interpret the copy number changes. Larger alterations were detected and validated by FISH, which were entirely recapitulated in the sequencing data. In contrast, the sequencing copy-number profiles described here revealed also smaller rearrangements, as well as copy-number neutral changes. Most of the copy number changes detected with single-cell sequencing were recapitulated in the integer copy-number profile obtained with the non-WGAed DNA extracted from the primary tumor, corroborating their authenticity. Exceptions include copy-number aberrations that are subclonal in origin (see below) and certain focal DNA imbalances, which could be true changes in the DTC or false positive artifacts from the WGA, requiring further validation. Calculation of SNP BAF-values provided the opportunity to examine allele-specific copy number changes and allowed validating a number of the DNA imbalances. Additionally, the SNP BAF-values highlighted important subclonal changes when comparing DTC with primary tumor.

Another issue seen in sequencing analysis of WGA products is obstructed alignment of reads containing true genomic sequence due to presence of WGA-adapter sequences in the reads. One could remove WGA-adaptors from amplicons through sonication before starting sequence library preparation of the cell’s WGA product (7), but even when using this approach we still detect several reads containing adaptor sequence. Thus it is important to trim off the adaptor sequences using informatic tools, before mapping single-cell sequence reads back to the human reference genome. An enzymatic solution to this problem may be developed.

The complexity of the genomic rearrangements was different between the two primary tumors, where MicMa083 was a highly aberrant tumor with a higher degree of subclonal changes. Both the primaries and DTCs showed gain of chromosome 1q, which is known to be a common event in ER positive breast cancers and is thought to occur at an early stage in tumor development (9, 33). MicMa003 and the corresponding DTC003 had 99% concordant copy-number profiles. MicMa003 did not show subclonal changes of significance and there was no evidence that the DTC had gone through further evolution at the copy-number level, which could indicate a less complex tumor progression where the dominating cell population within the tumor has gained the properties necessary for tumor cell dissemination. In contrast, the more highly aberrant DTC083 exhibited evidence of further evolution from its primary tumor. The tumor, MicMa083, had a highly subclonal copy-number profile and a lower general concordance to DTC083 (89%). In DTC083, cnLOH was observed where MicMa083 had a deleted copy of chromosome 13, and in addition a gain on chromosome 16p. These findings indicate that DTC083 may derive from a subclone of the primary that has undergone a subsequent duplication of the retained chromosome 13, and an allele-specific gain of 16p. DTC083 revealed trisomy 21, which does not appear to be subclonal in the primary tumor, and thus, could be a *de novo* gain or a small subclone not detected. If *de novo*, it may be either a stochastic event or a prerequisite for tumor dissemination, but a larger study is necessary for stating the relevance of the observation. Clonal events in the primary tumor were observed in DTC083, such as a putative chromothripsis (34) event on chromosome 2, as well as deletion and amplification of several other loci genome wide. These aberrations might be early copy-number events in tumor evolution. Although genomic changes are known to be important for tumor progression, they are not always directly transferrable to gene expression and phenotypic characteristics of the tumor cells. It is therefore important to develop methods that preserve the possibility for both DNA and RNA sequencing from single cells.

Naume et al. (35) found that presence of DTCs in BM resulted in reduced survival especially for breast cancer patients with tumors classified as ER positive, luminal A subtype (36). In our study the two presented patients had ER positive primary tumors and developed systemic relapse during follow up. Thus certain clones from the primary tumors, with quite different genomic complexity, seemed to be capable of metastasizing. Dissecting the molecular heterogeneity of the primary tumor and metastasis is further facilitated through deep sequencing (7, 9). To obtain further insight into the genomic pattern and differences between primary tumor and DTCs in breast cancer, larger number of patients needs to be analyzed, including all breast tumor subtypes. Also, higher coverage and investigation of multiple DTCs from the same patients, as well as their metastases will facilitate the study of the mechanism behind dissemination and recurrence. The feasibility of the method and analytical approach presented here opens the possibility to sequence not only the bulk tumor, but also single tumor cells from BM and blood.

## Author Contributions

Elen K. Møller wrote the manuscript with comments from all co-authors. Elen K. Møller and April Peterson produced the DTC sequencing libraries. Parveen Kumar and Thierry Voet did the single-cell sequencing data pre-processing and analysis. Jason Grundstad did, and assisted in, bioinformatical work. Elen K. Møller, with help from Silje Nord and Peter Van Loo, did aCGH and SNP-CGH data analysis. Randi R. Mathiesen, Renathe Fjelldal, Lars O. Baumbusch, Elin Borgen picked, amplified, and organized the DTC project together with Bjørn Naume, who funded it as well. Kevin P. White provided access to his lab and funded sequencing of the DTCs. Bjørn Naume, Anne-Lise Børresen-Dale, and Vessela N. Kristensen funded aCGH and SNP-CGH data production. Bjørn Naume, Anne-Lise Børresen-Dale, and Vessela N. Kristensen initiated the study and together with Silje Nord supervised the analysis.

## Conflict of Interest Statement

The authors declare that the research was conducted in the absence of any commercial or financial relationships that could be construed as a potential conflict of interest.

## Supplementary Material

The Supplementary Material for this article can be found online at http://www.frontiersin.org/Journal/10.3389/fonc.2013.00320/abstract

Figure S1 **Comparison of copy-number data of the DTCs generated from whole-genome sequencing (left side) and aCGH (right side)**. The piecewise constant fitting algorithm was used to generate segments with gamma = 25 and *k*_min_ = 5 for the sequencing data, and gamma = 60 and *k*_min_ = 25 for the aCGH data. **(A)** DTC003, **(B)** DTC083: genome-wide copy number changes show differences in detection sensitivity between sequencing and aCGH data.
