# Supplementary figures and images for: Next-Generation Sequencing of Disseminated Tumor Cells
Source: Front Oncol. 2013 Dec 31;3:320. doi: 10.3389/fonc.2013.00320 (PMC3876274; doi:10.3389/fonc.2013.00320)

**A**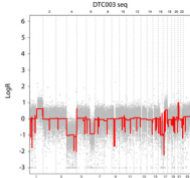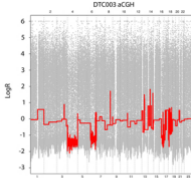**B**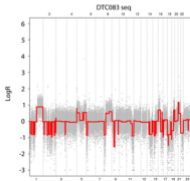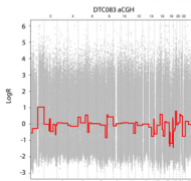

Supplement: Figure S1 — Comparison of copy-number data of the DTCs generated from whole-genome sequencing (left side) and aCGH (right side). The piecewise constant fitting algorithm was used to generate segments with gamma = 25 and kmin = 5 for the sequencing data, and gamma = 60 and kmin = 25 for the aCGH data. (A) DTC003, (B) DTC083: genome-wide copy number changes show differences in detection sensitivity between sequencing and aCGH data. [file 73302_Borresen-Dale_Presentation1.PDF]
